# Response to letter regarding “LASSO-derived model for the prediction of lean-non-alcoholic fatty liver disease in examinees attending a routine health check-up”

**DOI:** 10.1080/07853890.2024.2357228

**Published:** 2024-05-25

**Authors:** Chiao-Lin Hsu, Hsien-Chung Yu, Fu-Zong Wu

**Affiliations:** a Health Management Center, Kaohsiung Veterans General Hospital, Kaohsiung, Taiwan, Center for Geriatrics and Gerontology, Kaohsiung Veterans General Hospital, Kaohsiung, Taiwan; b Department of Radiology, Kaohsiung Veterans General Hospital, Kaohsiung, Taiwan, Faculty of Medicine, School of Medicine, National Yang-Ming University, Taipei, Taiwan, Department of Medical Imaging and Radiology, Shu-Zen Junior College of Medicine and Management, Kaohsiung, Taiwan

Dear Dr. Zhang and Dr. Li,

Thank you for your insightful comments and constructive critique of our research. We greatly appreciate the opportunity to address your concerns and provide further clarification.

Regarding the generalizability of our findings, we acknowledge the limitation posed by the single-centre, cross-sectional design of our study [1]. As you have suggested, we are actively pursuing collaborations with multiple institutions to expand our research to a multicentre, prospective, long-term follow-up approach. This will enable us to broaden our sample pool across diverse geographical regions and ethnic groups, thereby enhancing the robustness and generalizability of our findings.

Concerning the choice of regularization parameters and potential multicollinearity in our LASSO model, we recognize the validity of your critique. While the parameters included in our current study may not encompass all potential influencing factors, we are exploring the incorporation of additional biomarkers and genetic information to enrich our model and mitigate these concerns. Furthermore, we are investigating the integration of LASSO with other machine learning techniques to navigate complex feature interactions more effectively and potentially improve the model’s overall performance. In the future, we hope to integrate hospital electronic health records with the predictive model from our research to establish a Clinical Decision Support System (CDSS). This CDSS would serve as an auxiliary medical tool in clinical settings [2].

With respect to the accessibility of certain variables, such as visceral fat, for clinical application, we appreciate your perspective. In Taiwan, where our study was conducted, the measurement of visceral fat through body composition analysis is relatively accessible in many clinics and community screening programs. However, we acknowledge that this accessibility may vary across regions and countries, potentially limiting the model’s broader clinical application. To address this, we are actively exploring more cost-effective and convenient measurement techniques that could facilitate the adoption of our model in a wider range of clinical settings.

We understand your point regarding the similarity in predictive performance between our model and the Fatty Liver Index (FLI). While our study aimed to develop a large-scale prediction tool for clinical use, we recognize that there may be unknown factors or limitations that prevent our current model from surpassing the FLI's performance. This represents an area for further investigation and improvement in our future research endeavours.

We are grateful for your constructive feedback, which has provided us with valuable insights and a clear direction for enhancing our research. Your suggestions have highlighted areas for improvement, and we are committed to addressing these concerns through rigorous and collaborative efforts.

Sincerely

Chiao-Lin, Hsu, Hsien-Chung Yu and Fu-Zong Wu
